# 1544. HIV Following Oral Pre-Exposure Prophylaxis (PrEP) Initiation

**DOI:** 10.1093/ofid/ofad500.1379

**Published:** 2023-11-27

**Authors:** Aimee A Metzner, Guillaume Germain, François Laliberté, Alan Oglesby, Heidi Swygard, Sean MacKnight, Annalise Hilts, Mei Sheng Duh

**Affiliations:** ViiV Healthcare, Durham, North Carolina; Analysis Group, Montreal, Quebec, Canada; Groupe d'analyse, Montreal, Quebec, Canada; ViiV Healthcare, Durham, North Carolina; ViiV Healthcare, Durham, North Carolina; Analysis Group, Montreal, Quebec, Canada; Analysis Group, Montreal, Quebec, Canada; Analysis Group, Inc., Analysis Group, Inc., MA

## Abstract

**Background:**

Once-daily oral tenofovir-based combinations as pre-exposure prophylaxis (PrEP) are effective biomedical HIV prevention strategies. Still, low adherence and/or persistence can lead to decreased efficacy. This study describes the characteristics and HIV incidence in commercially-insured US oral PrEP users. Usage pattern results were previously presented.

**Methods:**

This retrospective study used IQVIA™ PharMetrics Plus data (1/1/2015–3/31/2020) to identify adults newly initiated (index date) on emtricitabine/tenofovir disoproxil fumarate (FTC/TDF) as daily PrEP. Users had ≥ 6 months (mos.) of continuous enrollment pre-index (baseline); those diagnosed with HIV or with antiretroviral therapy (ART) use during baseline were excluded. User characteristics were described during the baseline period. Users with both an HIV diagnosis and ART dispensing post-index were considered to have acquired HIV. A sensitivity analysis was conducted using ≥ 2 dispensings of ART on separate days to define HIV infection, regardless of documented HIV diagnosis. Time to HIV infection from the index date and from the latest PrEP dispensing was reported. A separate analysis without ≥ 6 mos. of continuous enrollment pre-index was performed.

**Results:**

In total, 24,232 FTC/TDF users were identified (**Table 1**). Mean [median] length of follow-up was 504 [390] days. By 3 mos. after initiation, 0.3% of FTC/TDF users had acquired HIV, which increased to 0.5% by 12 mos. (**Table 2**). The mean [median] time to detected HIV infection from index was 235 [95] days, and from the latest PrEP dispensing was 149 [29] days. 60.3% of FTC/TDF users with an HIV diagnosis had PrEP on hand at the time HIV was detected. In the sensitivity analysis requiring only ≥ 2 ART dispensings, rates were slightly higher (3 mos., 0.4%; 12 mos., 0.7%). In the analysis which removed the 6-month pre-index coverage requirement (**Table 3**), rates were also higher (3 mos., 2.2%; 12 mos., 2.5%).

**Figure:**
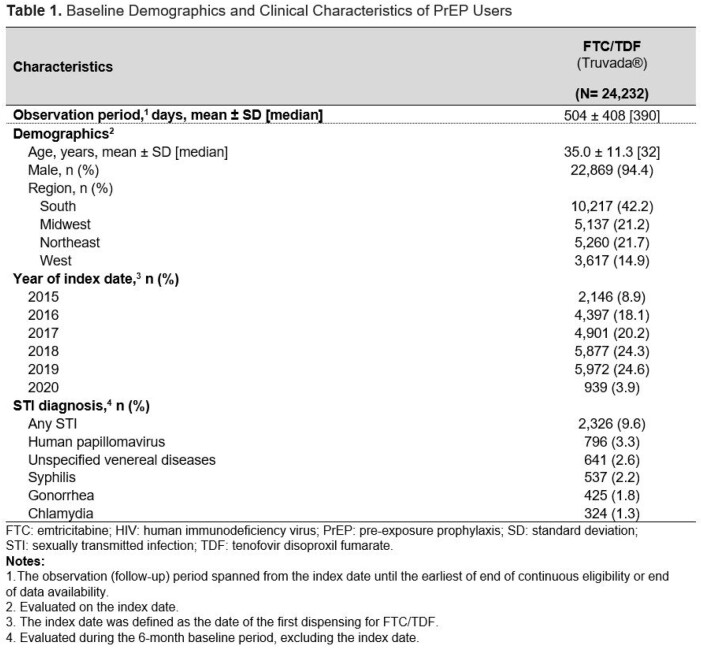

**Figure:**
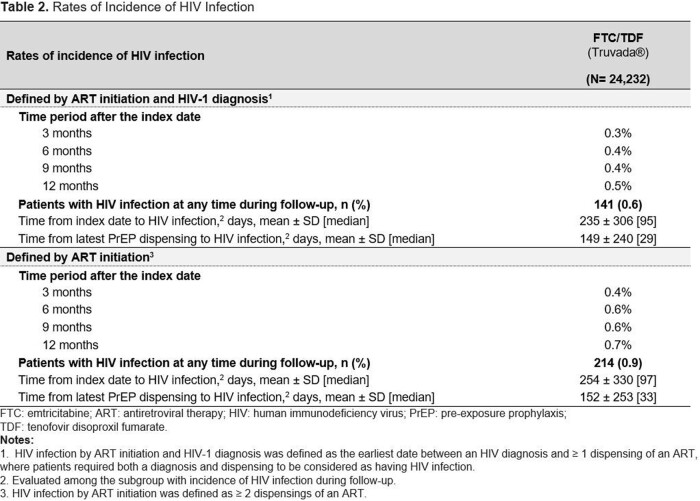

**Figure:**
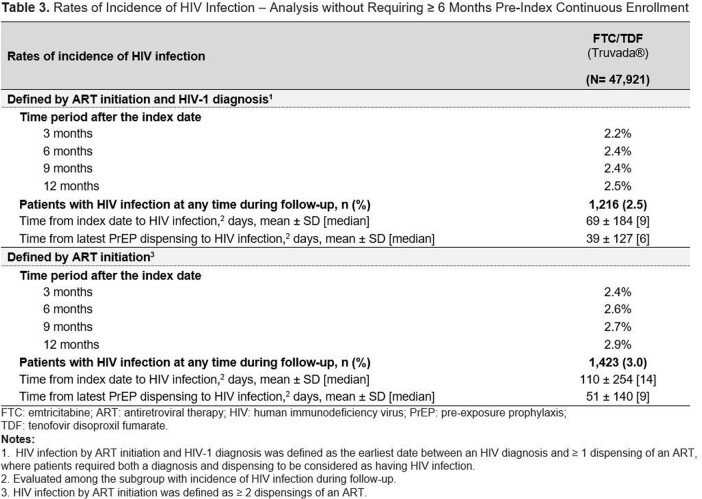

**Conclusion:**

HIV diagnosis following oral PrEP initiation is infrequent with higher incidence in those whose first covered healthcare encounter coincides with PrEP initiation. That a majority diagnosed with HIV had PrEP available at time of detection suggests challenges unexplained by access. Further research is needed to limit HIV acquisition despite access to oral PrEP.

**Disclosures:**

**Aimee A. Metzner, PharmD, AAHIVP**, ViiV Healthcare: Full-time employee (salary/benefits/etc.)|ViiV Healthcare: Stocks/Bonds **Guillaume Germain, MSc**, ViiV Healthcare: I am an employee of Analysis Group, a consulting company that received research funds from ViiV Healthcare to conduct this study. **François Laliberté, MS**, GSK: Grant/Research Support **Alan Oglesby, MPH**, GlaxoSmithKline: Employment|GlaxoSmithKline: Stocks/Bonds **Heidi Swygard, MD**, ViiV Healthcare: Employee of ViiV Healthcare|ViiV Healthcare: Stocks/Bonds **Sean MacKnight, MScPH**, ViiV: I am an employee of Analysis Group, a consulting company that received research funds from ViiV to conduct this study. **Annalise Hilts, BA**, ViiV (I am an employee of Analysis Group, a consulting company that received research funds from ViiV to conduct this study.): Grant/Research Support **Mei Sheng Duh, MPH, ScD**, Analysis Group, Inc.: Mei Sheng Duh is an employee of Analysis Group, Inc., a consulting company that received funding from GSK to conduct this study|ViiV Healthcare: Grant/Research Support

